# Mechanisms of inflammation in premature ovarian insufficiency and advances in therapeutic studies

**DOI:** 10.3389/fimmu.2026.1765073

**Published:** 2026-03-27

**Authors:** Yuling Chen, Ye Xu, Yue Lu, Wei Li, Fangfang Tao, Yuan Zhao

**Affiliations:** School of Basic Medicine, Zhejiang Chinese Medical University, Hangzhou, China

**Keywords:** autoimmune disease, follicular atresia, immunity, inflammation, premature ovarian insufficiency, therapeutic strategies

## Abstract

Premature Ovarian Insufficiency (POI) refers to the failure of ovarian function in women before the age of 40. Although the etiology of most cases remains unclear, accumulating evidence indicates that chronic inflammation plays a key role in the occurrence and development of POI. Patients with POI often exhibit phenomena such as imbalance of pro-inflammatory cytokines, abnormalities in the local ovarian immune microenvironment, and autoimmune ovarian inflammation. Inflammation may accelerate ovarian function decline through mechanisms such as inducing follicular cell apoptosis, follicular atresia, and tissue fibrosis. Several studies have shown that anti-inflammatory interventions, such as immunosuppressants, can modulate ovarian inflammation and thereby improve ovarian function. Given the potentially central role of inflammation in POI, this review focuses on the interplay between inflammation and POI. We first delineate the mechanisms by which inflammation drives POI, cataloguing the autoimmune diseases and inflammation-related biomarkers implicated in its pathogenesis. Subsequently, we summarize recent findings from multiple POI animal models (natural aging, chemotherapy-induced, autoimmune, genetic) and highlight emerging therapeutic strategies that target inflammatory pathways. Given the pronounced etiological heterogeneity of POI, not all patients exhibit a distinctly enhanced inflammatory profile. Consequently, targeted anti-inflammatory interventions have yet to be routinely adopted in clinical practice, and their broad efficacy in reversing ovarian dysfunction requires robust validation in prospective human trials. Nevertheless, as the multifaceted role of inflammation-whether as a primary driver or a secondary consequence-becomes clearer, mechanistically targeted immunomodulation may offer promising new avenues for POI management.

## Graphical Abstract

**Figure:**
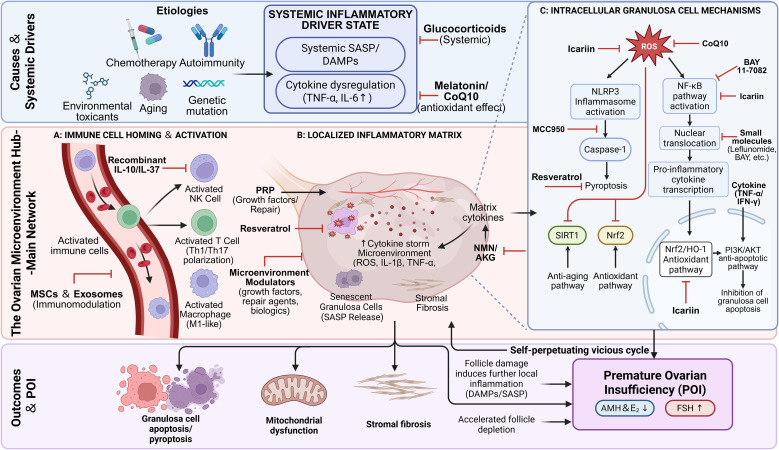
The integrated inflammatory network and therapeutic targets in POI. This schematic illustrates the multi-layered pathogenesis of POI and maps the corresponding etiology-specific intervention strategies. The top panel delineates systemic inflammatory drivers triggered by diverse initial insults. The middle panel depicts the localized ovarian microenvironment, highlighting immune cell homing, the extracellular cytokine storm, and the intricate intracellular molecular cascades—specifically the ROS-driven NLRP3 inflammasome and NF-κB pathways, alongside the protective Nrf2/SIRT1/PI3K-AKT axes—within granulosa cells. Ultimately, the figure demonstrates how these targeted interventions can concurrently neutralize oxidative stress, modulate immune responses, and halt the self-perpetuating vicious cycle of follicle-depleted ovarian inflammaging.

## 1 Introduction

POI is defined as ovarian failure in women before the age of 40 years, manifested by premature follicular depletion or dysfunction. Patients with POI often present with menorrhagia or scanty menstruation, hypergonadotropic and hypoestrogenic symptoms, which can severely affect fertility and increase the risk of osteoporosis and cardiovascular disease (1–3). Epidemiological data show that POI affects about 1 per cent of women under 40 years of age and that the cause is unknown in most cases (idiopathic in more than 70% of cases) (4).

The etiology of POI is highly heterogeneous and includes genetic factors, medical factors (e.g. chemo-radiotherapy), autoimmunity, and infections (5, 6). However, there is growing evidence that chronic inflammation plays a critical role in the development of POI. Patients with POI often have imbalances of pro-inflammatory cytokines, abnormalities in the local ovarian immune microenvironment, and autoimmune ovarian inflammation (7, 8). Inflammation may accelerate ovarian decline through mechanisms such as induction of follicular cell apoptosis, follicular atresia, and tissue fibrosis (7, 9). Inflammation is increasingly recognized as a central driver of POI. Therefore, systematically reviewing the inflammatory mechanisms and recent progress is indispensable for clarifying POI pathogenesis and identifying new therapeutic avenues.

Recognizing POI as a highly heterogeneous disorder, this review provides a targeted comparative analysis of the distinct inflammatory profiles across its various etiologies. To decode this complexity, we specifically delineate the causality of inflammation—exploring whether it acts as a primary pathogenic driver or a secondary consequence of follicular damage. Ultimately, these mechanistic insights serve as the foundation for a critical, evidence-based appraisal of emerging anti-inflammatory therapeutic strategies. In this paper, we will first review how immune cells such as macrophages and T cells accumulate in the ovary during POI and describe the signaling cascades and key inflammatory mediators involved, including the nuclear factor kappa B (NF-κB) and NOD-like receptor protein 3 (NLRP3) pathways together with pro-inflammatory cytokines like tumor necrosis factor alpha (TNF-α) and interleukin-6 (IL-6). Next, we discuss the effects of inflammation on follicular apoptosis and atresia, and the mechanisms of autoimmune-mediated ovarian damage. Then, we summarize the progress of basic and clinical research in the last five years, covering inflammation-related findings in multiple animal models of POI (natural aging, chemotherapy, autoimmunity, gene editing, etc.), as well as studies of inflammatory markers in human samples. Next, we critically evaluate POI therapeutic strategies targeting inflammation, including glucocorticoids, small molecule drugs, and regenerative approaches, and objectively sort out their mechanisms of action and research progress, and current limitations. Finally, we present the outlook based on the summary of existing studies, with a view to providing a robust framework for future POI mechanism studies and personalized clinical interventions.

## 2 Mechanisms of inflammation in POI

### 2.1 Involvement of immune cells

The ovary is not a completely immune-exempt organ, and immune cells are involved in normal ovarian physiological processes (e.g. local inflammatory response at ovulation). During the development of POI, there are abnormal changes in the composition of immune cells and their functional status that contribute to the pathological inflammatory response of the ovary (10).

Macrophages are important immune cells in the ovary and play a dual role in tissue homeostasis and inflammation. Under normal conditions, M1/M2 maintains a dynamic balance in the ovary (11), Within the inflammatory milieu of POI, however, there is often an over-activity of the M1 type and an under-activity of the M2. This leads to a flood of pro-inflammatory factors and a weakening of the mechanisms that inhibit inflammation (12–14). Some animal studies have confirmed that inflammation and tissue damage in POI models can be attenuated by increasing the proportion of M2 macrophages in the ovary (15). For example, after transplantation of placental mesenchymal stem cells (PMSC) into a rat model of POI, a decrease in localized interleukin-1 beta (IL-1β), TNF-α, etc. an increase in IL-10, and a significant increase in CD206-positive macrophages, an M2 markers, were observed in the ovary (15). It indicates that modulation of macrophage polarization status can influence the inflammatory process of POI.

T cells also play an important role in the immunopathology of POI. Th cells can differentiate into different subtypes, each secreting different cytokines that influence the intensity and nature of the immune response (16–18).

IL-12 and interferon-gamma (IFN-γ) are two crucial cytokines for T helper type 1 cell (Th1) differentiation. T-bet serves as the master regulator for inducing IFN-γ production and Th1 cell differentiation.

The differentiation of T helper type 2 cells (Th2) is induced by interleukin-4 (IL-4) GATA3 is the master transcription factor for Th2 differentiation. It can directly bind to various regulatory regions within the IL-4 and interleukin-13 (IL-13) gene loci. This action not only positively feedbacks to promote its own expression but also further amplifies the production of cytokines such as IL-4 (19).

In POI, elevated Th1- and Th17-type cytokines are frequently detected, suggesting hyperactive auto-inflammatory responses that drive cell-mediated tissue damage (7). Conversely, impaired regulatory T cell (Treg) function fails to maintain immune tolerance, leaving these auto-reactions unchecked (20). This T-cell imbalance synergizes with B-cell-mediated autoantibody production to exacerbate ovarian injury. Notably, in specific subtypes such as autoimmune POI, cytotoxic T lymphocytes (CTLs) directly target ovarian antigens, further accelerating follicular depletion (21). In the peripheral blood of POI patients, an expanded population of plasma cell-like B cells has been reported (22), likely reflecting persistent autoantigenic stimulation.

Innate immune cells, particularly natural killer (NK) cells and macrophages, also exhibit POI-specific alterations. Recently, a single-cell sequencing data reveals a concomitant decrease in the proportions of NK cells and classical monocytes in the peripheral blood of POI patients, contrasting with the elevated ratios of B cells and CD4/CD8 T cells (23). This suggests a unique alteration in the peripheral immune profile of patients with POI: NK cells and monocytes/macrophages may be peripherally hypofunctional or depleted or migrate locally to the ovary in large numbers; whereas adaptive immunity (B cells, Th cells) is relatively active (22). In addition, the altered neutrophil/lymphocyte ratio in patients with POI was found to reflect the dynamic adjustment of innate and adaptive immunity (7).

A critical paradox in the immunopathology of POI lies in the compartmental distribution of natural killer (NK) cells. While human ovarian biopsies are clinically rare, evidence from human follicular fluid analyses and murine models of autoimmune oophoritis consistently demonstrates the active recruitment and accumulation of NK cells within the damaged ovaries (24). To reconcile this apparent discrepancy, we postulate that the peripheral depletion does not indicate a systemic loss or hypofunction of innate immune cells. Instead, it reflects an active, targeted compartmental shift driven by a local chemotactic gradient. In the pathological context of POI, the inflamed or senescent ovarian stroma likely secretes high levels of specific chemokines (such as CXCL10/IP-10), which act as a powerful ‘sink’ to actively recruit circulating NK cells out of the bloodstream and into the targeted tissue. Once localized in the ovary, these recruited NK cells exacerbate follicular apoptosis and microenvironmental disruption through the concentrated release of IFN-γ and cytotoxic granules (e.g. perforin and granzyme) (23, 25). Thus, the peripheral reduction of NK cells is not an isolated immunodeficiency, but rather a direct systemic reflection of aggressive, localized immune cell recruitment to the site of ovarian injury.

**Figure 1:**
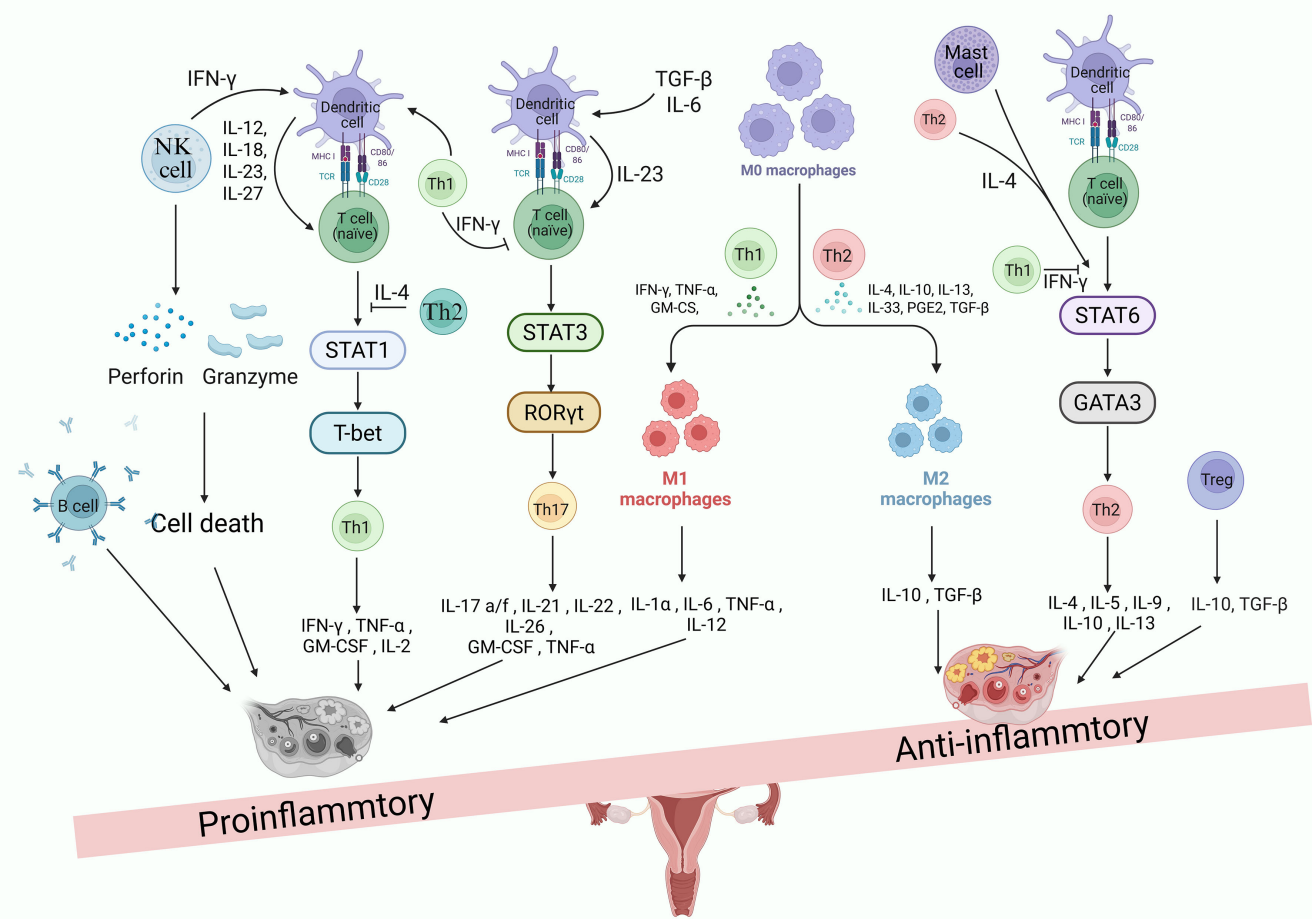
The imbalance of immune cells and immune factors leads to POI. The inflammatory microenvironment of POI is collectively shaped by a variety of immune cells: macrophages, T cells, B cells, and NK cells, which perform their respective functions or interact with one another, forming a chronic inflammatory network. If this network shifts in a pro-inflammatory and destructive direction—such as excessive activation of M1 with insufficient M2; hyperactive Th1 and Th17 responses or migration and cytotoxic activity of NK cells—it will drive the occurrence and progression of POI. Conversely, promoting the restoration of its balance (e.g. enhancing the roles of anti-inflammatory M2 macrophages and regulatory T cells) may slow down the disease process.

Collectively, the inflammatory microenvironment of POI is shaped by a variety of immune cells: macrophages, T cells, B cells, and NK cells each in their own way or interacting with each other to form a chronic inflammatory network. If this network is shifted in a pro-inflammatory and destructive direction, it will drive the development of POI; conversely, if it can be brought back into balance (e.g. by promoting the role of anti-inflammatory M2 macrophages and regulatory T cells), the disease process may be slowed down (**Figure 1**).

### 2.2 Activation of inflammatory signaling pathways

Among the inflammatory mechanisms associated with POI, aberrant activation of inflammatory pathways such as the nuclear factor kappa-B (NF-κB) signaling pathway and NOD-like receptor thermal protein domain associated protein 3 (NLRP3) inflammasome has attracted much attention. NF-κB is a classical pro-inflammatory transcription factor, and its activation induces the expression of a variety of cytokines and chemokines to drive inflammatory responses (26, 27). In ovarian tissues, inappropriate NF-κB activation is thought to promote chronic inflammation and immune damage. It has been noted that the NF-κB pathway tends to be highly expressed during ovarian aging, which is accompanied by increased levels of pro-inflammatory factors (28). Elevated NF-κB activity has also been observed in animal models of POI, such as increased nuclear translocation of the NF-κB p65 subunit in ovarian tissues, accompanied by up-regulation of TNF-α, IL-1β, etc. (15, 29). Abnormal activation of NF-κB may be triggered by a variety of upstream signals, e.g. ovarian localized pattern recognition receptors (PRRs) recognizing hazardous signals such as damage-associated molecular pattern (DAMPs) can activate the inhibitor of κB kinase (IκK) complex, releasing NF-κB into the nucleus to initiate inflammatory gene transcription (30). This chronic low-grade NF-κB-mediated inflammation is thought to be involved in the accelerated decline of ovarian function (28).

**Figure 2:**
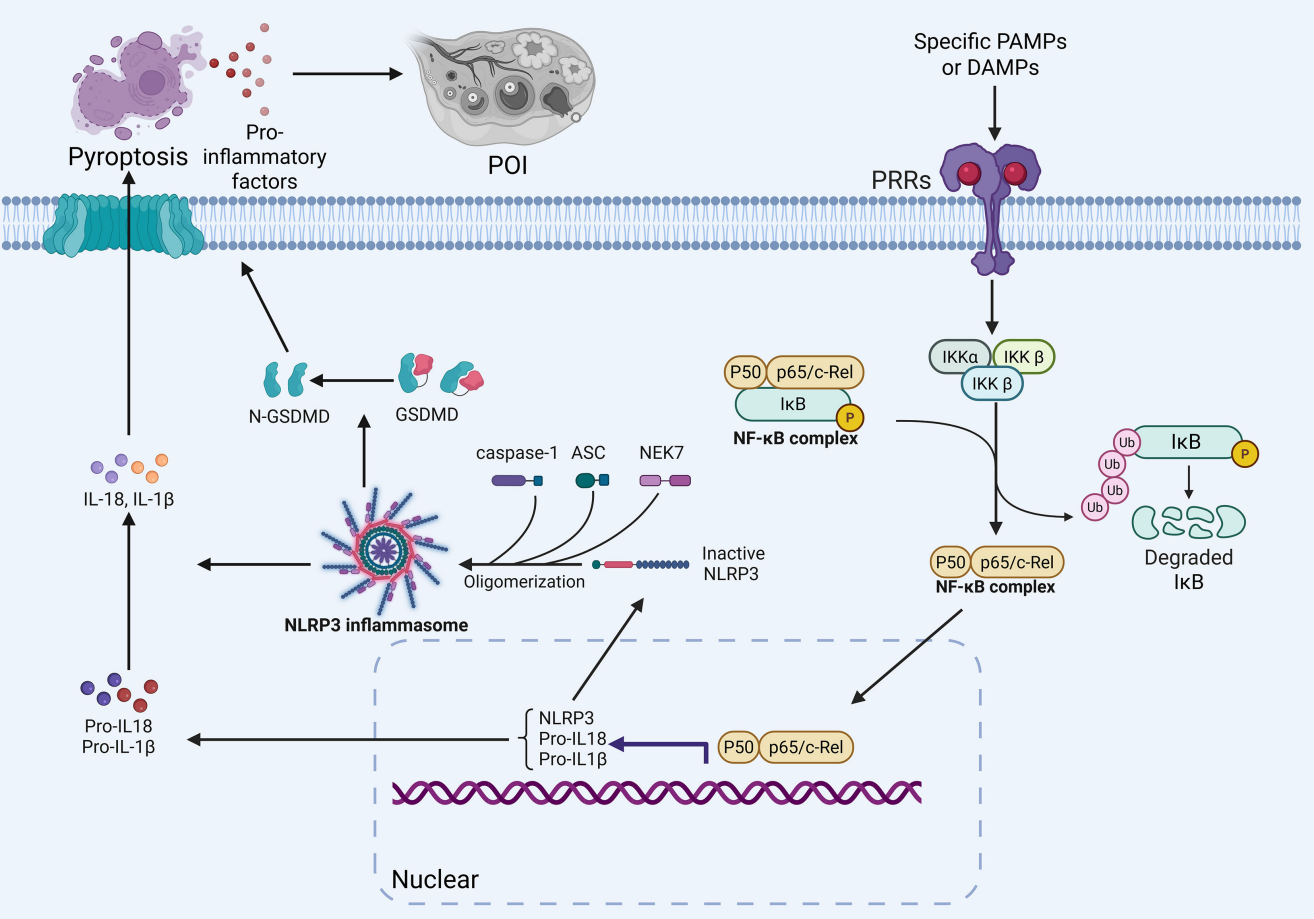
Activation of the NF-κB and NLRP3 inflammatory pathways promotes the occurrence of POI. In POI, inflammatory pathways such as NF-κB and NLRP3 are abnormally activated, and they synergistically promote ovarian inflammatory responses and tissue damage, which is one of the important driving forces behind ovarian insufficiency. Excessive activation of inflammasomes can induce pyroptotic death of ovarian granulosa cells, release more pro-inflammatory mediators, and create a vicious cycle of amplified local ovarian inflammation.

In addition, NLRP3 inflammasome has been a hotspot of POI research in recent years. NLRP3 inflammasome is an important component of intrinsic immunity that senses the activation of intracellular danger signal assembly, which leads to caspase-1 cleavage and release of mature IL-1β and IL-18, triggering inflammatory cell necrosis (i.e. pyroptosis) (31–33). It has been proposed that NLRP3 inflammasome-mediated pyroptosis may be one of the key mechanisms of ovarian injury in POI (15). Increases in NLRP3 and its downstream products were found in both ovarian aging and POI models: for example, levels of NLRP3 protein and activated caspase-1 and IL-1β were elevated in mouse ovaries with age, suggesting an accumulation of ‘aseptic’ inflammation with age (34, 35). NLRP3 is predominantly expressed in the cytoplasm of granulosa cells, follicles and oocytes, and is highly active in aging and damaged ovaries (36). Over-activation of inflammatory vesicles induces pyroptotic death of ovarian granulosa cells, releasing more pro-inflammatory mediators, resulting in a vicious cycle of localized inflammatory amplification in the ovary. In summary, aberrant activation of inflammatory pathways such as NF-κB and NLRP3 in POI, which synergize with each other to promote ovarian inflammatory responses and tissue damage, is one of the key drivers of ovarian insufficiency (15). These pathways also provide us with potential targets for intervention (**Figure 2**).

### 2.3 Imbalance of inflammatory factors

Patients with POI often show an imbalance between pro-inflammatory and anti-inflammatory cytokines. Studies have shown that patients with POI have increased serum levels of several pro-inflammatory cytokines, such as TNF-α, IL-6, interleukin 8 (IL-8), IL-17, IFN-γ, whereas levels of the anti-inflammatory cytokine, IL-10, are significantly lower than normal (7, 37). This imbalance of elevated pro-inflammatory factors/decreased anti-inflammatory factors suggests the presence of a chronic inflammatory microenvironment in patients with POI. Excessive pro-inflammatory factors may directly damage ovarian tissues: for example, TNF-α can activate an apoptotic cascade via its receptor (38, 39), inducing programmed cell death in follicular granulosa cells and stromal cells; IL-6 and others can inhibit follicular cell survival signaling pathways and induce oxidative stress, which can exacerbate the damage to ovarian function (40–42). On the contrary, the reduction of anti-inflammatory factors such as IL-10 weakens the negative feedback regulation of inflammation by the body and further amplifies the inflammatory response (43–45). It is to be noted that some of the cytokine changes may be more complex. For example, a multiprotein antibody array analysis found that the expression of 11 proteins including IL-17 family members (IL-17F, IL-17C) and IFN-γ receptor 1 was downregulated in the serum of patients with POI, whereas another metabolic factor, Afamin, was upregulated (46).

The above results suggest that the inflammatory factor profile associated with POI is multidimensional, with elevated typical pro-inflammatory factors and some possible disorders of immunomodulatory factors. Overall, the imbalance of inflammatory factors creates an unfavorable microenvironment for ovarian function and poses a threat to follicular survival. These inflammatory factor imbalances provide important clues to our understanding of the pathophysiology of POI (**Figure 1**).

Another critical conceptual challenge in deciphering the cytokine profiles of POI is determining the precise causality of the inflammatory response: is it the primary driver of ovarian damage, or a secondary consequence of follicular depletion? The answer largely depends on the specific etiology of the disease (47). In autoimmune POI, aberrant immune activation acts as the initiating driver, where primary cytokine storms (e.g. elevated TNF-α and IFN-γ) directly induce granulosa cell apoptosis and disrupt follicular maturation (20, 48).

Conversely, in chemotherapy-induced or idiopathic POI, initial toxic insults or accelerated atresia led to the massive accumulation of apoptotic and senescent cells (29). This accumulation triggers the release of DAMPs and senescence-associated secretory phenotypes (SASP), which subsequently recruit innate immune cells to the ovary (10, 49). In these contexts, the inflammatory cytokine milieu is initially a secondary consequence—a physiological clearance mechanism that pathologically overshoots. Ultimately, regardless of the initiating trigger, this dynamic evolves into a self-perpetuating vicious cycle: primary follicular damage induces secondary inflammation, which in turn acts as a driver to further accelerate the depletion of the remaining ovarian reserve (8, 41).

### 2.4 The effect of inflammation on follicular apoptosis and atresia

Follicular apoptosis and atresia are part of the dynamic balance of follicles in the ovary, but in POI, excessive or premature follicular apoptosis and atresia is one of the direct causes of ovarian reserve failure. The chronic inflammatory state accelerates follicular apoptosis and atresia through several mechanisms: first, pro-inflammatory cytokines directly induce follicular cell death. For example, TNF-α binding to its receptor activates apoptotic signaling pathways (e.g. caspase-8/3 cascade) in ovarian granulosa cells, leading to apoptosis of granulosa cells and oocytes, thus preventing follicles from continuing to develop and prematurely atresia (20, 30). Inflammatory mediators also impair follicular cell viability by inducing oxidative stress and mitochondrial dysfunction. Secondly, inflammation disrupts pro-survival signals required for follicular development. Some vital survival pathways such as phosphoinositide 3-kinase (PI3K)/protein kinase B (AKT) and mammalian target of rapamycin (mTOR) are inhibited in the chronic inflammatory environment, whereas the expression of pro-apoptotic factors (e.g. BAX) is upregulated, disrupting the balance between follicular survival and apoptosis (36). Third, inflammasome-mediated pyroptosis has devastating effects on the follicular pool. It has been shown that activation of NLRP3 inflammasome during ovarian ageing leads to pyroptotic death of a large number of follicular granulosa cells, which in turn causes premature follicular atresia (9, 36). Activated NLRP3 inflammasome not only triggers local inflammation by releasing IL-1β, etc. but also triggers tissue remodeling processes such as fibrosis, creating a scarred environment that is not conducive to follicular survival (15, 40). For instances, NLRP3 overactivation was strongly associated with ovarian interstitial fibrosis and follicle reduction in the POI rat model (15).

In addition, the ovarian microenvironment provides nutritional support and signaling transport for follicular growth and development, ovulation and corpus luteum formation, and the close interaction between the follicle and the ovarian microenvironment determines the fate of the follicle, and thus the lifespan of the ovary (47, 50). Chronic inflammation can also impair ovarian vascularity and stromal supply, deteriorating the follicular supportive environment and thus indirectly promoting atresia (51, 52).

**Figure 3:**
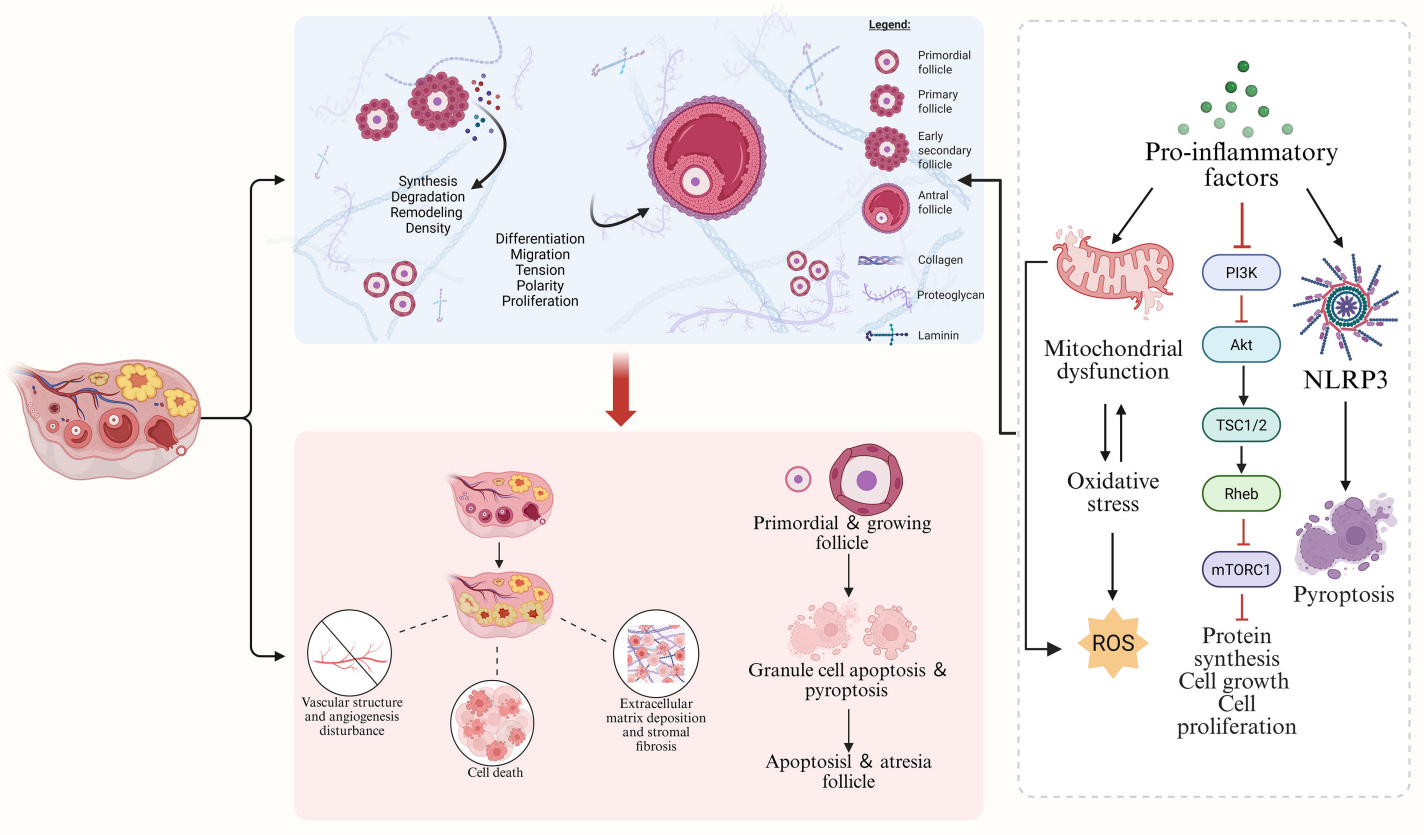
The impacts of inflammation on the ovary. Chronic inflammatory states accelerate follicular apoptosis and atresia through multiple mechanisms: First, pro-inflammatory cytokines induce oxidative stress and mitochondrial dysfunction, which lead to follicular cell death and impair follicular cell viability; second, inflammation disrupts the pro-survival signals required for follicular development; third, activation of the NLRP3 inflammasome causes pyroptotic death of a large number of granulosa cells in follicles, which in turn leads to premature follicular atresia; in addition, chronic inflammation can also damage ovarian blood vessels and stromal supply, deteriorating the supportive environment for follicles and thereby indirectly promoting follicular atresia.

Overall, inflammation accelerates the process of follicular apoptosis and atresia through a dual pathway of direct killing of follicular cells and destruction of the follicular microenvironment. This explains why follicular depletion is significantly faster than physiological apoptosis in autoimmune ovarian inflammation or post-chemotherapy inflammatory states. Inhibition of the aberrant inflammatory pathway is expected to slow down excessive follicular apoptosis and protect ovarian reserve (**Figure 3**).

### 2.5 Autoimmune-mediated inflammation of the ovary

Autoimmunity is one of the most significant mechanisms leading to POI, and about 4%-30% of POI cases are thought to be autoimmune-related (53) (Latest research tends to estimate between 4 and 15 per cent (54)). Autoimmune POI usually presents as an attack on the ovarian tissue by the body’s immune system, leading to autoimmune ovarian inflammation (oophoritis), which in turn impairs follicular function (2, 55). Histologically, autoimmune ovarian inflammation is characterized by the presence of lymphocytic and plasma cell infiltration in the ovary, mainly around the follicular membrane cells of the developing follicle and the corpus luteum, whereas primordial and primary follicles are often ‘spared’ (53). This selective mode of attack means that the immune system primarily targets cells in the ovary that express specific antigens (e.g. steroid synthesis-associated cells), while early follicles may temporarily survive. Clinically, autoimmune POIs often co-exist with other autoimmune diseases, particularly adrenal, autoimmune polyglandular syndrome (APS) (56) and thyroid disease (53). A study has demonstrated that the prevalence of severe autoimmune diseases in women with POI is over twice that of the general female population, and the incidence of such diseases continues to increase for more than ten years following the diagnosis of POI (57). In many patients, anti-ovarian antibodies or steroid cell antibodies can be detected, the latter targeting ovarian steroid synthases such as 21-hydroxylase (21-OH), 17α-hydroxylase (17-OH), or P450 side-chain cleavage enzymes (P450scc). These autoantibodies suggest that the immune system misidentifies ovarian endocrine cells as ‘non-self’ and thus mediates immune attack.

POI frequently co-occurs with various systemic autoimmune diseases, suggesting a shared genetic and immunological basis. **Table 1** summarizes the prominent peripheral cytokine dysregulations strictly characterized in these highly concurrent autoimmune disorders. While comprehensive cytokine profiling exclusively within the rare ‘overlap cohorts’ (patients concurrently diagnosed with both the specific autoimmune disease and POI) remains sparse in the current literature, accumulating evidence indicates that the fundamental inflammatory mediators driving these systemic diseases—particularly IL-6, TNF-α, and IFN-γ—are similarly upregulated within the ovarian microenvironment of POI patients (7, 20, 46). This extensive mechanistic parallel implies that POI might share a universal ‘pro-inflammatory cytokine signature’ with its autoimmune comorbidities, warranting future targeted cross-disease investigations.

**Table 1:** Immunologic markers of POI-associated autoimmune diseases.

Autoimmune diseases	Elevated cytokines	Reduced cytokines	Autoantibodies	Reference
Autoimmune Thyroiditis (AT)	CXCL10, IL-1β, IL-6, IL-8, IL-12, TNF-α, IL-17/IL-22, IL-10	TGF-β	TPO-Ab	(144–146)
Type 1 Diabetes (T1D)	IL-1β, IL-6, IL-8, IL-9, IL-10, IL-17, IL-33, TNF-α,	IL-4, IFN-γ	TPO-Ab, IA-2A, IAA, ICA, TG-Ab	(147–150)
Addison’s Disease(AD)	CXCL10, CXCL11, IL-6, IL-17A, COX-2,	IL-10	21OHAb, 17OHAb, P450sccAb, StE-Abs	(53, 151–153)
Systemic lupus erythematosus (SLE)	IL-1, IL-6, IL-10, IL-17, IL-18, IL-21, IFN-α, TNF-α	IL-2	ANA, Anti-dsDNA, anti-RBP	(154–156)
Rheumatoid Arthritis (RA)	TNF-α, IL-1α, IL-1β, IL-6, IL-17A, IL-12, GM-CSF, CXCL10, MCP-1, CX3CL1, IL-10		ACPAs	(157–160)
Sjögren’s Syndrome (SS)	IFN-γ, IL-12, IL-18, TNF-α, IL-1β, IL-6, IL-17, IL-21, BAFF, APRIL, CXCL13, MCP-1	TGF-β, IL-4	ANA, ENA, anti-HMGB1 antibody,	(161–163)
Graves’ Disease (GD)	IL-17A, IL-22, IL-6, CXCL-10, ICAM-1, IL-10	CCL19	TRAb, TPO-Ab, ANA, Anti-dsDNA	(164–168)
Vitiligo	IFN-γ, IL-1β, IL-4, IL-17A, IL-13, TNF-α,	IL-10	TPO-Ab, TG-Ab,	(169–173)
Coeliac Disease (CeD)	IL-2, IFN-γ, IL-21, IL-6, IL-17A, IL-22, TNF-α, IL-10, CXCL9, IL-8, CCL2, CCL20		anti-TG, EMA	(174–177)
Selective IgA Deficiency (sIgAD)	IL-12, IL-4, IL-5, IL-13, IL-17A, IL-6, IL-8, TNF-α, TNF-β, IFN-γ		anti-IgA	(178, 179)
Crohn’s disease	IL-1β, IL-18, IL-17A, IL-23, IL-12, TNF-α, IL-6, GM-CSF	IL-10	pANCA, Anti-pancreas antibody	(180–183)

Systematically catalogues the autoimmune diseases commonly associated with POI, along with the corresponding cytokine profiles exhibiting up- or down-regulation and the autoantibody specificities frequently encountered in clinical practice.

However, there is a lack of specific and reliable *in vitro* diagnostic indicators to confirm the autoimmune etiology of POI (53). Immunological studies have shown that in autoimmune ovarian inflammation, Th cells (especially Th1 and Th17) and CTL may be directly involved in the ovarian attack (16–18); autoantibodies produced by B cells may also damage ovarian tissues through complement-mediated cytotoxicity (23). In addition, innate immune cells such as macrophages and NK cells are involved in the expansion of autoimmune inflammation. Chronic tissue destruction as a result of autoimmune inflammation can contribute to a dramatic decrease in ovarian reserve over a short period of time. Of interest, autoimmune-mediated ovarian damage may be partially reversible in the early stages: some cases have been reported in which menstruation and some ovarian function can be restored in such patients by applying immunosuppressive therapies such as glucocorticoids (53). In the mechanism of POI, autoimmune inflammation provides an extreme example that emphasizes the profound influence of the immune system on ovarian function.

## 3 Advances in ovarian inflammation in natural ageing and various POI models

In recent years, numerous basic and clinical studies have been conducted in different models around the relationship between inflammation and POI, ranging from animal experiments, *in vitro* cellular models to patient samples analyses, and important advances have been made. In the following, new findings related to inflammation will be reviewed according to different types of POI research models. Meanwhile, to fully appreciate the etiological heterogeneity of the disease, **Table 2** provides a comprehensive comparative analysis of the distinct inflammatory signatures—including key cytokines, immune cell dominances, and underlying signaling pathways—across autoimmune, chemotherapy-induced, genetic, and idiopathic POI subtypes.

**Table 2:** Comparative inflammatory profiles across distinct etiologies of POI.

Etiology	Role of inflammation	Key dysregulated cytokines	Dominant immune cells	Primary signaling pathways & mechanisms	Reference
Idiopathic/Aging-related POI	Vicious Cycle/Mixed (Chronic “Inflammaging”)	↑ Basal IL-6, TNF-α, IL-18	Shift towards pro-inflammatory M1 Macrophages, altered NK cell cytotoxicity	SIRT1 downregulation, chronic ROS-mediated NF-κB hyperactivation, accumulation of senescent cells (SASP).	(8, 10, 36, 41)
Chemotherapy-Induced POI	Secondary Consequence (Pathological over-clearance)	↑ IL-1β, IL-6, IL-18, TNF-α, ↓ IL-10	Recruited Macrophages (scavengers), Natural Killer (NK) cells	DAMPs/SASP release, excessive ROS generation, NF-κB activation, NLRP3 inflammasome pyroptosis, DNA damage response (p53).	(27, 29, 79, 142)
Autoimmune POI	Primary Driver (Initiates follicular damage)	↑ IFN-γ, TNF-α, IL-17, IL-6 ↓ IL-10, TGF-β	Autoreactive CD4+/CD8+ T cells, B cells (autoantibodies), M1 Macrophages	Th1/Th17 polarization, classic NF-κB activation, impaired Treg immunotolerance.	(20, 94, 97, 143)
Genetic/Chromosomal POI	Secondary Consequence	↑ IL-6, TNF-α (during atresia)	Resident Macrophages (cleaning apoptotic bodies)	Altered TGF-β superfamily signaling, accelerated intrinsic apoptosis, mitochondrial dysfunction.	(110–113)
	(Follows accelerated atresia)	↓ BMP15, GDF9			

Provides a comprehensive comparative analysis of the distinct inflammatory signatures across autoimmune, chemotherapy-induced, genetic, and idiopathic POI subtypes, synthesized from recent mechanistic advancements.

### 3.1 Ovarian inflammation associated with natural ageing

Natural physiological aging also leads to diminished ovarian function, and the process of ovarian senescence is usually characterized by a decline in the quality and number of oocytes or follicular pools, which is similar to POI in terms of outcome but with a different time course. Mechanisms of ovarian senescence include, but are not limited to, telomere shortening (58–60), mitochondrial dysfunction (61, 62), oxidative stress (63, 64), DNA damage (65, 66), apoptosis and autophagy (67–69). Subsequent research has identified common inflammatory mechanisms between ovarian senescence and POI.

The term ‘inflammaging’ is used to denote the chronic low-grade inflammation that occurs with age and is considered to be one of the hallmarks of organismal ageing (70). A parallel phenomenon has been observed in the ovary: with age, the level of pro-inflammatory factors in the ovarian tissue gradually increases, and the activity of inflammatory pathways is enhanced, possibly accelerating follicular depletion. In a study of ovarian ageing in mice, it was reported that the ovaries of aged mice exhibited a tendency towards elevated expression of NLRP3 inflammasome and an augmentation in downstream inflammatory mediators, such as interleukin 8 (IL-18) (49). Accordingly, *Nlrp3* knockout mice maintained ovarian function during aging better than the wild type: knockout mice had significantly higher levels of anti-Müllerian hormone (AMH) and more follicles in ovarian tissue at 12 months of age (36). This indicates that inhibition of inflammatory vesicles can delay ovarian aging.

Further mechanistic investigations revealed that DNA damage repair pathway genes were up-regulated in the ovaries of Nlrp3-deficient aged mice, slowing down the genetic changes associated with oocyte ageing. Thus, the accumulation of inflammation may drive ovarian aging by exacerbating DNA damage and apoptosis. In humans, a study comparing ovarian tissues from women of different ages found that the expression of inflammation-related genes (e.g. *IL-6, TNF*) was elevated and the expression of anti-inflammatory factors (e.g. *IL-10*) was lowered in the ovaries of the older age group, which supports a shift from an anti-inflammatory to a pro-inflammatory local microenvironment in ovaries (36, 71).

Single-cell sequencing technology has also provided new insights: a single-cell study of follicular fluid from young, old and POI women found altered immune cell composition and enhanced underlying inflammatory signaling in the follicular microenvironment of both POI patients and older women (1). For instance, the number of γδ -T cells were lower in both the POI and advanced age groups than in the normal fertile age group, while certain inflammatory and endoplasmic reticulum stress-related pathways were more enriched in their follicular microenvironment cells. Interestingly, the study also revealed the presence of vascular endothelial growth factor A (VEGFA)- fms-related tyrosine kinase 1 (FLT1) interactions between monocyte macrophages and granulosa cells in the normal group, whereas this protective signaling was absent in the POI and advanced age groups, which may have led to a deterioration of the follicular supportive environment and to ‘hyperinflammation’. This discovery suggests that aging and POI may share certain inflammation-driven pathological processes, such as dysregulated communication between follicular support cells.

**Figure 4:**
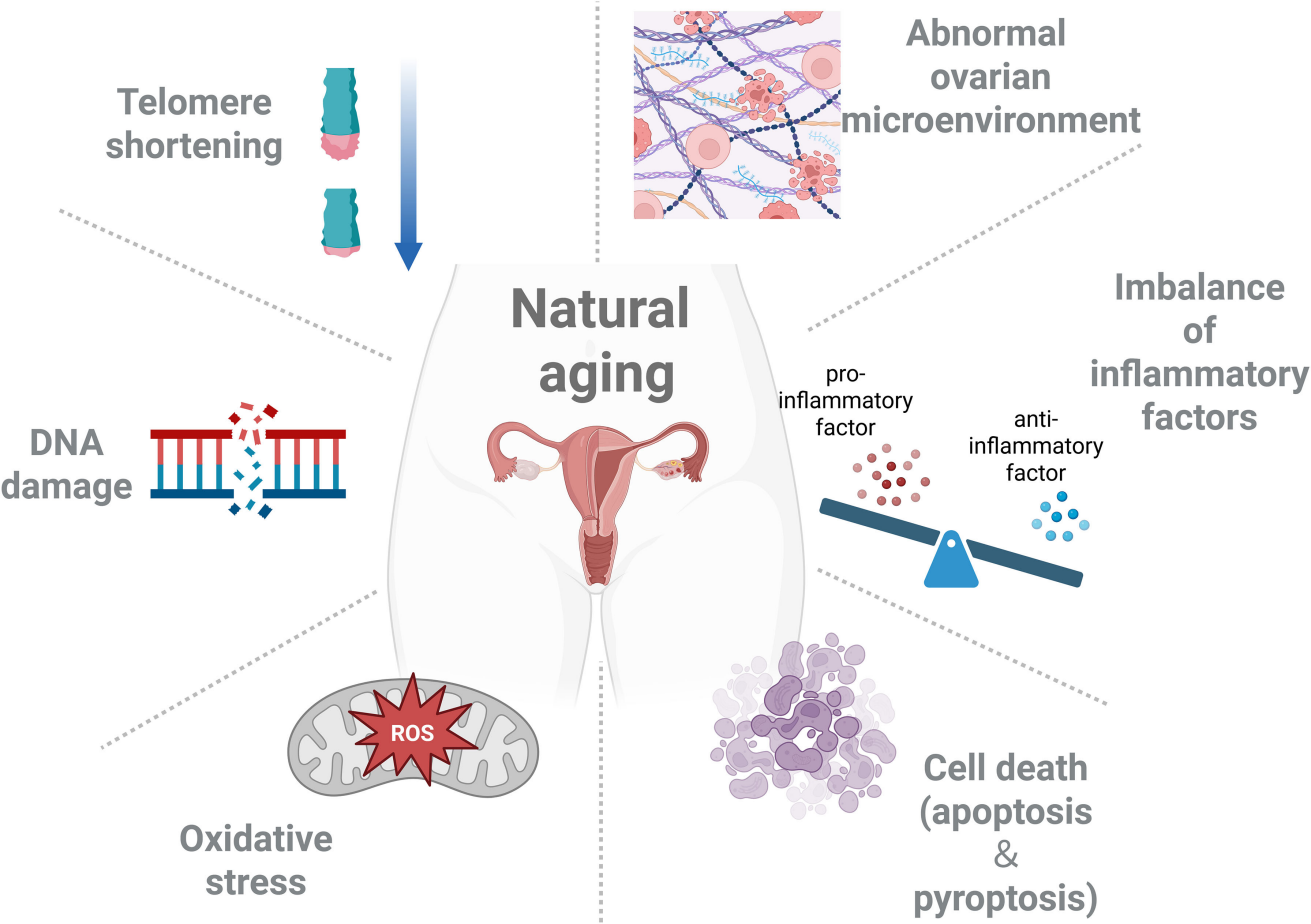
The mechanism of natural aging of the ovaries. Natural physiological aging can also lead to the decline of ovarian function. The mechanisms related to ovarian aging include, but are not limited to, telomere shortening, mitochondrial dysfunction, oxidative stress, DNA damage, cell apoptosis, and autophagy.

Overall, comparing natural ovarian aging with POI reveals that inflammation is both part of the aging process and a factor that is prematurely and excessively activated in POI. Uncovering this commonality could help us to draw on anti-aging anti-inflammatory strategies in response to POI. For example, studies are now focusing on the possibility of prolonging the reproductive lifespan using anti-inflammatory drugs: drugs targeting NLRP3, TNF-α, etc. have shown some promise in slowing the decline of ovarian function (36). Consequently, it is theoretically and practically useful to consider POI as ‘premature inflammatory aging of the ovaries (**Figure 4**).

### 3.2 Inflammation studies in a chemotherapy-induced POI model

Oncological chemotherapeutic agents (e.g. cyclophosphamide, cisplatin, etc.) can cause ovarian toxicity and induce secondary POI (72, 73). Chemotherapy-induced ovarian damage is not only attributed to direct DNA damage, but is also closely associated with secondary inflammatory responses (74, 75). In animal models, cyclophosphamide (CTX)-induced POI is widely used to study inflammatory mechanisms and interventions. In the CTX-induced mouse model of POI, ovarian tissue showed a significant inflammatory response: up-regulation of the expression of pro-inflammatory cytokines such as IL-1β, IL-6 and TNF-α, activation of inflammatory signals such as NF-κB, and detectable follicular cell pyknotoxicity and apoptosis (40, 76, 77). For example, it has been reported that cisplatin chemotherapy resulted in dramatic up-regulation of *IL-1β*, *IL-6*, *TNF-α* mRNA and down-regulation of *IL-10* in rat ovaries, suggesting that the ovaries were placed in a strongly pro-inflammatory environment (78). Based on this mechanism, many anti-inflammatory drugs have shown protective effects in chemotherapeutic POI models. For example, NLRP3 inflammasome is regarded as a key node of chemotherapy-induced ovarian cell death, and the application of NLRP3 inhibitors significantly improves ovarian function. A study found that supplementation with nicotinamide mononucleotide (NMN) inhibited NLRP3-mediated ovarian granulosa cell death and attenuated CTX-induced damage to ovarian reserve in mice (79). Meanwhile, α-ketoglutarate (AKG) protects granulosa cells from pyroptosis and restores follicular viability by inhibiting the activation of the NLRP3/Caspase-1 pathway in a POI model (80). These results validate the pathogenic role of the NLRP3 inflammasome-caspase-1 axis in chemotherapeutic POI and suggest that increasing cellular nicotinamide adenine dinucleotide (NAD^+^) levels or metabolic modulation could be an intervention strategy.

In addition to NLRP3 targeting, other anti-inflammatory and antioxidant agents have shown efficacy in chemotherapeutic POI models. Melatonin, a strong antioxidant with anti-inflammatory properties (81–83), has been extensively studied in cyclophosphamide- and cisplatin-induced POI. Animal studies have shown that administration of melatonin effectively reduces chemotherapy-induced oxidative stress and pro-inflammatory factors in the ovary, and reduces DNA damage and apoptosis in follicular cells (84, 85). In a rat study of cisplatin-induced POI, melatonin significantly inhibited the elevation of p-NF-κB, TNF-α, and IL-1β and upregulated anti-inflammatory markers such as IL-10 in ovarian tissue (40). Mechanistically, melatonin exerts a broad-spectrum anti-inflammatory effect by blocking the entry of NF-κB into the nucleus and reducing the expression of cyclooxygenase-2 (COX-2) and inducible nitric oxide synthase (iNOS). Eventually, the follicle survival rate and fertility of animals in the melatonin-treated group were significantly higher than those in the untreated group (40). Natural products such as celastrol (86) and resveratrol (14) have also been used to protect ovaries from radiotherapy damage due to their anti-inflammatory and antioxidant properties. It has been shown that resveratrol inhibits inflammatory signals and increases antioxidant enzyme levels in the ovaries and attenuates radiation-induced premature ovarian failure (87). In summary, in the chemotherapy-induced POI model, a large body of evidence supports the key role of inflammation in the injury process: inhibition of inflammatory mediators (e.g. IL-1β, TNF-α) or blockade of inflammatory pathways (e.g. NF-κB, NLRP3) both partially reversed the ovarian functional impairment. This provides an idea for fertility preservation in tumor patients, i.e. the combination of anti-inflammatory or antioxidant agents along with chemotherapy is expected to reduce ovarian side effects.

**Figure 5:**
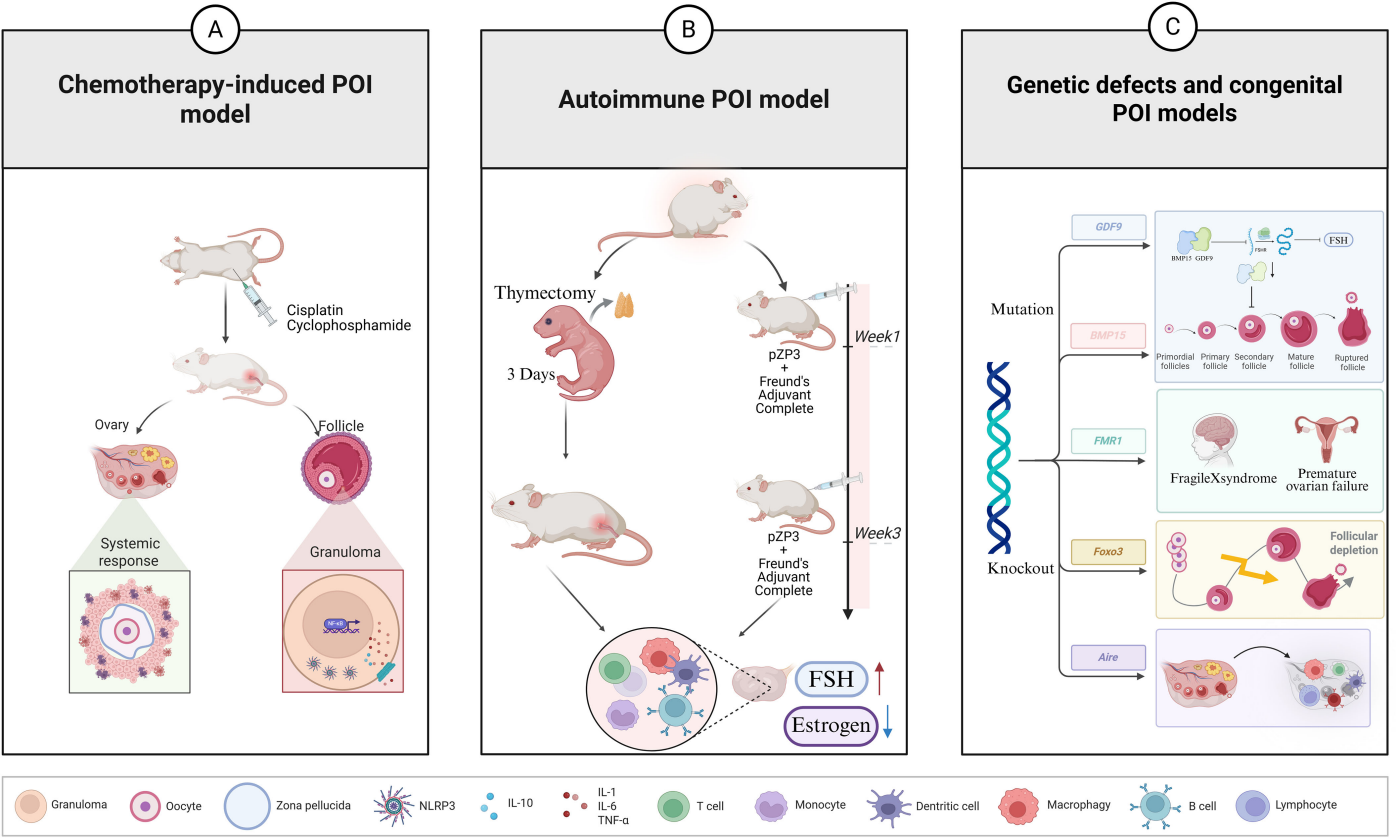
Various animal models of POI in recent years. **(A)** Chemotherapy-induced POI models: Chemotherapeutic drugs such as cyclophosphamide and cisplatin are often injected intraperitoneally to induce premature ovarian failure models. **(B)** Autoimmune POI models: Thymectomy on the 3rd day after birth in newborn mice (3d-Ntx) or immunizing mice with ZP3 peptides combined with adjuvants can induce anti-ovarian autoantibodies and ovarian lymphocyte infiltration in mice, both of which can trigger autoimmune ovarian inflammation and follicular destruction. **(C)** Genetic defects and congenital POI models.

It is important to note that ovarian fibrosis represents a further manifestation of ovarian ageing subsequent to chemotherapy, with inflammation playing a pivotal role in the development of fibrosis (88–90). The release of pro-inflammatory factors, such as TGF-β1, has been demonstrated to induce fibroblast activation and matrix overdeposition in the aftermath of damage to ovarian tissue (40, 42, 48, 91). The persistent activation of NLRP3 inflammasome has also been suggested to promote the process of ovarian fibrosis (9, 92). Therefore, anti-inflammatory treatment might not only protect follicular cells, but might also preserve the structural integrity of ovarian tissue by inhibiting fibrosis. Overall, the chemotherapy-associated POI model clearly supports the concept of ‘inflammation leads to ovarian damage’ and provides valuable information for screening potential therapeutic targets (e.g. NF-κB, NLRP3, etc.) and interventional drugs (**Figure 5**).

### 3.3 Advances in autoimmune POI modelling

As outlined in the Mechanisms section above, autoimmunity plays a significant role in POI. In order to reproduce this process experimentally, researchers have developed several animal models of autoimmune POI. The classic model is the experimental autoimmune oophoritis (EAO) model. The following methods are commonly used: immunizing mice against ovary-specific antigens (e.g. follicular membrane protein, Zona Pellucida peptide, ZP3, etc.), or performing a thymectomy on day 3 in neonatal mice (3d-Ntx). Both of these methods trigger autoimmune ovarian inflammation and follicular destruction (93, 94). For example, immunization of mice with ZP3 peptide fragments in combination with adjuvants induced the production of anti-ovarian autoantibodies and ovarian lymphocyte infiltration, leading to folliculopenia and estrogen decline over a period of weeks, mimicking the POI phenotype (95–97). In these models, a massive infiltration of T cells, B cells and macrophages in the ovary is observed, accompanied by stagnant follicular development and impaired luteinization. In addition, thymectomy (removal of Treg development) in 3-day-old mice also leads to autoimmune ovarian inflammation in adulthood, as evidenced by diffuse lymphocytic infiltration of the ovary and persistently high levels of follicle-stimulating hormone (FSH), similar to POI in humans (93, 94).

These models provide a platform for the testing of immunosuppressive therapies. For instance, in autoimmune POI mice, interventions such as the application of CTLA4-Ig fusion proteins (which block T-cell co-stimulation) or cyclophosphamide (a high-dose immunosuppressive regimen, different from the low-dose regimen that caused the POI) reduced ovarian inflammation and improved ovarian function (53). It has been shown that high-dose glucocorticoids partially restore follicular development and reduce autoantibody titers in an animal model of autoimmune POI (98). This is consistent with clinical case reports of glucocorticoids prompting patients to resume menstruation (99). In addition, biologics targeting specific immune pathways are beginning to be tried in animal models, e.g. IL-17 neutralizing antibodies for blocking the Th17 pathway (100, 101), B-cell activating factor (BAFF)/a proliferation-inducing ligand (APRIL) inhibitors to reduce the B-cell drive (102, 103). In the course of conducting tissue analyses of the models in question, the investigators discovered that, in addition to classical lymphocyte infiltration, innate immune mechanisms such as inflammatory vesicles may also be involved in tissue destruction. For instance, the activation of NLRP3 and IL-1β was observed in ovarian infiltrating macrophages, indicating a combination of innate and adaptive immunity (8, 41, 104). In conclusion, the study of autoimmune POI models consolidates the causal role of inflammation, especially that triggered by autoimmunity, on ovarian failure and provides an experimental basis for immunomodulatory therapy (**Figure 5**).

### 3.4 Findings in models of genetic defects and congenital POIs

Genetic factors have been demonstrated to account for a significant proportion of the pathogenesis of POI. A number of knockout or mutant mice have been found to exhibit a phenotype similar to that of POI, thus providing a valuable tool for investigating the molecular mechanisms underlying this condition. While the predominant defect in numerous genetic POI models is located in the genes that regulate follicular development itself, some models also demonstrate alterations in immunity and inflammation. For instance, autoimmune regulator (*Aire*) knockout mice are susceptible to autoimmune polyglandular diseases, including ovarian inflammation, due to a lack of central immune tolerance. This can be considered a model of a genetic defect inducing immune-mediated POI (105). These mice exhibit symptoms consistent with ovarian lymphocyte infiltration and follicular destruction from an early age, thus providing a robust validation of the role of *AIRE* mutations in human APS-1 combined POI. Moreover, the transcription factor forkhead box protein O3 (FOXO3) has been demonstrated to play a pivotal role in the regulation of cellular stress turnover, cell survival, and cell death/senescence (106, 107). While its primary function is as an intrinsic reproductive endocrine mechanism that results in premature follicular depletion due to persistent activation of the primary follicular pool in *Foxo3* knockout mice, secondary inflammatory and fibrotic alterations have been observed in the ovaries of mice following follicular depletion. The premature menopausal state itself has been shown to induce low-grade chronic inflammation, which is analogous to the state of ‘inflammatory senescence’ (108).

There are few genetic POI models that focus directly on inflammation. However, analysis of specimens from patients with mutations causing POI suggests immune involvement. For instance, patients with full mutations in fragile X messenger ribonucleoprotein 1 (*FMR1*) (Fragile X-associated POI) frequently exhibit co-morbidities with autoimmune diseases (109, 110). Historically, phenotypic analyses of growth differentiation factor 9 (*Gdf9*) and bone morphogenetic protein 15 (*Bmp15*) knockout mice have predominantly focused on the morphological arrest of follicular development—such as impaired granulosa cell proliferation and follicular blockade at the primary stage—leaving the local inflammatory milieu largely uncharacterized (111, 112). Although direct inflammatory profiling of ovaries from these mouse models and patients with homologous mutations remains limited, recent evidence from mutant zebrafish models provides critical mechanistic insights (113). These studies demonstrate that BMP15 deficiency alone is sufficient to act as an upstream trigger, directly activating ovarian macrophages and precipitating pathogenic inflammation. Coupled with the prevalent imbalance between pro-inflammatory and trophic factors observed in human POI, it is plausible to postulate a dual-hit mechanism in mammalian genetic POI: BMP15/GDF9 dysfunction not only intrinsically arrests folliculogenesis but also secondarily amplifies the local ‘inflammatory network’ by compromising the immunosuppressive ovarian microenvironment, thereby accelerating follicular depletion.

A further study identified substantially modified expression profiles of inflammatory and immune-related genes in the ovaries of patients with POI via integrative gene screening and transcriptome analysis. This finding indicates that the inflammatory pathway may still be implicated in downstream tissue responses even if the primary defect is in developmental genes (36). Moreover, mice exhibiting mutations in pathways associated with premature aging (e.g. DNA damage response) manifest premature ovarian failure accompanied by ‘sterile inflammation’. This observation lends further support to the notion that chronic inflammation constitutes a prevalent mechanism underlying premature tissue aging. Overall, genetic and congenital models of POI emphasize the direct impact of genetic defects on ovarian function, while recent histological studies have begun to reveal common inflammatory features across different genotypes of POI. This provides a basis for considering inflammation as a universal link in the pathogenesis of POI in the future (**Figure 5**).

## 4 Inflammation-related therapeutic strategies and research advances

To effectively counteract the complex inflammatory network in POI, contemporary therapeutic strategies have evolved from conventional hormone replacement to targeted immunomodulation and regenerative medicine. This section critically evaluates emerging anti-inflammatory interventions and highlights both their mechanistic promise and current translational limitations. A comprehensive summary of these therapeutic agents, including their specific targets, utilized models, and current clinical trial statuses, is provided in **Table 3**.

**Table 3:** Summary of inflammation-targeted therapeutic strategies in POI.

Therapeutic strategy/agent	Molecular/cellular targets	Experimental model/evidence source	Key outcomes	Clinical trial status & limitations	Reference
Glucocorticoids	Suppresses autoantibody production; Reduces T cell & macrophage infiltration	Human case reports; Autoimmune POI animal models	Resumption of menses in selected autoimmune cases; Rescues ovarian function;	Highly Restricted: Discouraged for routine use due to severe adverse effects (e.g. osteopenia); lacks large-scale RCTs.	(53, 98, 114–117)
CoQ10	Neutralizes ROS; Blocks ROS-mediated NF-κB inflammatory cascade	Human RCTs; Chemotherapy-induced POI/aging animal models	Preserves follicle pool; Improves oocyte quality and mitochondrial energetics	Clinical Application: Established adjuvant therapies in reproductive clinics; highly effective in breaking the ROS-inflammation vicious cycle.	(120–123)
Small Molecule Inhibitors (BAY 11-7082, MCC950, NMN)	Specific blockade of IκB phosphorylation (NF-κB) or NLRP3 inflammasome assembly	*In vitro* GC models; Chemotherapy-induced POI animal models;	Reduces GC inflammatory factor release; Prevents NLRP3-mediated pyroptosis	Preclinical/Experimental: Strong mechanistic proof-of-concept; requires further *in vivo* validation and pharmacokinetic profiling in POI.	(36, 79, 118, 119, 124)
Recombinant Cytokines & Growth Factors (IL-10, IL-37, VEGF)	Systemic inflammatory balance; FLT1 receptor agonism	Preclinical POI models; Human plasma correlative studies	Promotes local neoangiogenesis; Rebalances systemic and local immune responses	Preclinical Phase: Theoretical and *in vivo* animal support; direct clinical trials for POI are currently lacking.	(71, 126–130)
Stem Cell Therapy (e.g. MSCs & Exosomes)	Paracrine immunomodulation; Secretion of anti-inflammatory cytokines	Chemical toxicant-induced, Chemotherapy-induced POI models; Genetic & aging models	Attenuates stromal fibrosis; Promotes neoangiogenesis & follicular survival	Experimental Phase: Promising pilot data, but heavily bottlenecked by safety concerns, transient survival, and lack of standardization.	(15, 74, 131–135)
Platelet-Rich Plasma (PRP)	Growth factors (VEGF, TGF-β); Microenvironment repair	Human POI/DOR patients (*in vivo*)	Counteracts chronic inflammation; ‘Reawakens’ dormant primordial follicles	Observational/Pilot: Limited to observational human data; routine application remains premature pending placebo-controlled RCTs.	(136–140)
Natural Phytochemicals (Resveratrol, Icariin)	Activates SIRT1 & PI3K/AKT; Inhibits NLRP3 & NF-κB; Promotes Treg expansion	Human follicular fluid; Preclinical autoimmune/Chemotherapy-induced POI models	Downregulates IL-1β, TNF-α, IL-6; Mitigates oxidative stress; Inhibits GC apoptosis	Mixed: Resveratrol has translational success in RCTs; ICA shows robust multi-target pleiotropy but remains in the preclinical phase.	(14, 87, 141–143)

Summary of emerging inflammation-targeted therapeutic strategies for Premature Ovarian Insufficiency (POI), detailing their mechanistic targets, preclinical/clinical evidence, and current translational limitation.

### 4.1 Glucocorticoids and broad-spectrum immunosuppression

Glucocorticoids (e.g. prednisone, dexamethasone) are potent anti-inflammatory immunosuppressants. Glucocorticoid therapy has a long history of use in autoimmune-related POIs. Some early case reports suggest that high-dose glucocorticoids may restore menstruation and some endocrine function in patients with autoimmune ovarian inflammation (53, 98). Glucocorticoids are able to reduce the inflammatory response of the ovary in all aspects by inhibiting the activation of many inflammatory cells and cytokine gene transcription. For example, it reduces lymphocyte infiltration, lowers autoantibody titers, and inhibits macrophage secretion of IL-1, TNF, etc. (114).

In autoimmune POI animal models, the administration of hormone therapy has been shown to significantly alleviate the destruction of ovarian tissue induced by autoimmunity (115). However, the efficacy of glucocorticoid therapy exhibits significant inter-individual variability, and its prolonged utilization has been associated with adverse effects, including osteoporosis and an increased risk of infection (116, 117). There exists no uniform guideline recommending its use for POI. However, short-term, full-dose glucocorticoid therapy may be considered as an experimental measure in patients with POI who have clear evidence of autoimmunity (e.g. comorbid adrenal or thyroid autoimmune disease, presence of ovarian autoantibodies) (53).

In addition to glucocorticoids, other immunosuppressive agents such as cyclophosphamide (utilized as an immunosuppressive agent at high doses), tacrolimus and intravenous immunoglobulin have been employed in individual cases of POI, albeit with limited evidence supporting their use. In conclusion, broad-spectrum immunosuppressive strategies targeting the immune-mediated component of inflammation appear to be effective in patients with POIs of well-defined immunological etiology. However, these strategies require rigorous screening for indications and risk trade-offs. As the mechanisms of inflammation in POI are better understood, more targeted immunomodulatory therapies are emerging as alternatives to nonspecific suppression with traditional hormones.

### 4.2 Small molecule therapies targeting inflammatory pathways

The development of small molecule drugs targeting specific inflammatory signaling cascades represents a significant emerging frontier in POI therapy. Extensive evidence highlights the pathogenic role of the NF-κB and NLRP3 inflammasome pathways in POI, driving considerable interest in their respective inhibitors. Among NF-κB inhibitors, experimental agents like BAY 11-7082 (an IκB phosphorylation inhibitor) have demonstrated efficacy in reducing granulosa cell inflammation and apoptosis *in vitro* (118, 119). Clinically utilized immunosuppressants, such as azathioprine and leflunomide, may also attenuate ovarian inflammation through NF-KB blockade. Notably, naturally occurring small molecules with established safety profiles, such as Coenzyme Q10 (CoQ10), offer a highly translatable approach to targeting this cascade. By actively neutralizing reactive oxygen species (ROS) within the ovarian microenvironment, CoQ10 effectively blocks the ROS-mediated activation of NF-κB, thereby protecting granulosa cells from autoimmune and toxicological damage and preserving the remaining follicle pool (120, 121). Importantly, this mechanistic rationale has been successfully translated into clinical practice. Recent large-scale meta-analyses and randomized controlled trials confirm that CoQ10 supplementation in women with diminished ovarian reserve (DOR) or impending POI significantly improves follicular developmental competence, mitigates oxidative stress, and enhances overall clinical pregnancy outcomes (122, 123).

Of the NLRP3 inflammasome inhibitors, MCC950 is a highly selective inhibitor of NLRP3 inflammasome, which has been validated to be effective in a variety of models of inflammatory diseases (124, 125). The utilization of MCC950 in ovarian ageing models has been reported to diminish IL-1β levels in the ovary and preserve a greater number of follicles (36, 49). While direct evidence in POI models remains limited, other small molecules like NMN and AKG operate through similar mechanisms to inhibit NLRP3-mediated pyroptosis (79, 80).

Beyond classical signaling inhibitors, rebalancing the systemic inflammatory state using recombinant anti-inflammatory cytokines (e.g. IL-10 or interleukin 37, IL-37) holds therapeutic promise (126, 127). Furthermore, given the recent study suggesting a causal association between reduced plasma vascular endothelial growth factor (VEGF) and POI pathogenesis (71), exogenous VEGF supplementation or FLT1 receptor agonism may concurrently promote local neo angiogenesis and modulate immune responses (128). Finally, repurposing rheumatological biologics-such as anti-TNF-α (e.g. infliximab) and anti-IL-6 receptor antibodies (e.g. tocilizumab) monoclonal antibodies-to sequester specific pro-inflammatory cytokines represents a compelling avenue for future research (129, 130). Although recent case reports document the successful use of anti-TNF therapy in refractory autoimmune diseases, its precise efficacy and safety in autoimmune POI necessitate rigorous prospective validation.

### 4.3 Mesenchymal stem cells in POI: paracrine immunomodulation and current limitations

It is worth noting that stem cell and exosome therapies, although not traditional ‘small molecules’, work in large part through paracrine anti-inflammatory factors, which can be discussed here. MSCs transplantation demonstrates significant improvement in ovarian function in an animal model of POI (15, 131, 132). Mechanistic studies have revealed that MSCs are able to secrete large amounts of anti-inflammatory cytokines (e.g. IL-10, TGF-β) and induce macrophage conversion to the M2 phenotype, thereby creating a reparative immune microenvironment. For example, transplantation of placenta-derived MSCs into POI rats resulted in a decrease in IL-1β and TNF-α and an increase in IL-10 and IL-4, and a significant decrease in NLRP3 inflammasome expression in the ovaries, as well as a restoration of follicle number and hormone levels (15). This evidence suggests that stem cells achieve therapy by modulating innate immune responses (e.g. inhibiting TLR4/NF-κB signaling) and adaptive immunity (increasing Treg ratios, etc.). MSCs-secreted exosomes have a similar function and are rich in anti-inflammatory miRNAs and proteins that cross the Blood-Follicle Barrier to modulate local inflammation in the ovary (74).

While stem cell-based therapies—particularly those utilizing MSCs—have demonstrated substantial regenerative and immunomodulatory potential in preclinical models, their clinical translation is heavily bottlenecked by several critical limitations. Foremost among these are safety concerns, including the theoretical risk of tumorigenicity (especially concerning embryonic or induced pluripotent stem cells), ectopic tissue formation, and unpredictable local immune reactions (133). Furthermore, the long-term efficacy of transplanted stem cells remains highly debated. Current evidence suggests that their survival within the fibrotic and inflamed ovarian microenvironment is often transient (133, 134); therapeutic benefits appear to rely primarily on short-lived paracrine effects (via secretomes and exosomes) rather than sustained cellular engraftment (133, 135). Finally, the field suffers from a severe lack of standardization. The pronounced heterogeneity in cell sources, optimal culturing protocols, delivery routes (systemic vs. targeted intraovarian injection), and precise dosing regimens makes it exceedingly difficult to ensure reproducible clinical outcomes. Consequently, until rigorous, large-scale prospective clinical trials resolve these standardization and safety hurdles, stem cell therapy for POI must be regarded as an experimental endeavor.

### 4.4 Platelet-rich plasma: an emerging regenerative strategy for ovarian microenvironment repair

PRP is an autologous blood concentrate rich in essential growth factors (including VEGF, PDGF, and TGF-β) and immunomodulatory cytokines. Given the complex inflammatory microenvironment characteristic of POI, intraovarian injection of autologous PRP has recently emerged as a compelling regenerative strategy. Mechanistically, local administration of PRP is hypothesized to counteract chronic ovarian inflammation, promote neoangiogenesis, and remodel the fibrotic stroma, thereby facilitating the ‘reawakening’ of dormant primordial follicles (136, 137).

However, a critical appraisal of the current clinical evidence is imperative. While numerous pilot studies and small prospective cohort trials have reported promising short-term outcomes—such as transient improvements in ovarian reserve markers (AMH, antral follicle count, AFC), resumption of menses, and occasional spontaneous pregnancies—these findings are predominantly derived from observational human data without robust, placebo-controlled groups (137–140). High-quality, large-scale randomized controlled trials (RCTs) are currently lacking. Therefore, while PRP represents a biologically plausible intervention to modulate the immune microenvironment in specific POI subtypes, its routine clinical application remains premature pending definitive validation from prospective human data.

### 4.5 Traditional Chinese medicine and natural phytochemicals

Compared to conventional immunosuppressants, Traditional Chinese Medicine (TCM) and natural phytochemicals offer a unique advantage in POI management due to their multi-target pleiotropy and favorable safety profiles. Rather than broadly suppressing the immune system, these agents primarily act by fine-tuning the local inflammatory microenvironment and alleviating oxidative stress. At the molecular level, specific natural compounds have demonstrated robust anti-inflammatory efficacy in preclinical POI models. For instance, resveratrol, a natural polyphenol, exerts its protective effects by activating the SIRT1 pathway, which subsequently inhibits the assembly of the NLRP3 inflammasome and downregulates the secretion of IL-1β and IL-18 in granulosa cells (14, 87, 141).

Similarly, icariin (ICA), a primary active flavonoid extracted from *Epimedium*, has been shown to significantly attenuate immune-mediated ovarian damage. Mechanistically, ICA downregulates the classic NF-κB signaling cascade, thereby reducing the localized accumulation of TNF-α and IL-6, while simultaneously inhibiting granulosa cell apoptosis via the PTEN/AKT/mTOR/AMPK pathway (142). Furthermore, in murine models of autoimmune POI, ICA ameliorates ovarian structural damage and restores function by activating the Nrf2/HO-1/Sirt1 signaling pathway and promoting local regulatory T cell (Treg) expansion (143).

Crucially, while many natural compounds remain in the preclinical testing phase, several well-defined pure monomers have successfully crossed the translational gap into human clinical trials for POI and diminished ovarian reserve (DOR). Building on its robust preclinical promise, resveratrol has been extensively evaluated in RCTs. Clinical data confirm that dietary supplementation with this compound effectively downregulates the expression of pro-inflammatory cytokines (such as IL-6 and TNF-α) in human follicular fluid and mitigates oxidative stress via SIRT1 activation. The successful clinical translation of such specific monomers provides a promising blueprint for the future development of other preclinical candidates like ICA.

## 5 Conclusion and future directions

A substantial body of research evidence suggests that inflammation plays an integral role in the development of premature ovarian failure. Whether it is an imbalance of pro-inflammatory cytokines, aberrant activation of inflammatory signaling pathways, or immune cell-mediated autoimmune ovarian destruction, premature follicular apoptosis and depletion can lead to POI (7, 53). Inflammation may be both a common downstream pathway for different etiologies of POI (e.g. chemotherapeutic injury, autoimmunity, genetic mutations, etc.) and a self-amplifying factor that exacerbates and maintains ovarian decline. Consequently, considering POI as an inflammation-driven disease provides us with a new perspective for integrating various etiological factors. Within this framework, the success of various types of anti-inflammatory interventions in POI models is encouraging. These include glucocorticoids, which are used to suppress autoimmune inflammation; small molecules, which are used to block the release of inflammatory mediators; stem cell therapies, which are used to remodel the immune microenvironment; and multi-targeted modulation of natural products from traditional Chinese medicine. The efficacy of these strategies in reducing the inflammatory load of the ovary is evidenced by the restoration of ovarian function. In the future, the introduction of anti-inflammatory concepts in POI treatment is expected to open up new pathways.

Yet, there is still a lot that remain to be investigated. Firstly, the POI patient population is highly heterogeneous. It is unknown whether the inflammation is comparable across subtypes. For example, there is a clear lack of large studies to address what proportion of patients with idiopathic POI have significant inflammatory abnormalities. In the future, for patients with ‘inflammation-dominant’ POIs, screening biomarker such as cytokine profiles, immune cell subtypes, inflammation-related gene expression, etc. will be indispensable.

Secondly, inflammation is a wide category, and in particular for POI, we need to further refine what inflammatory pathways/factors are ‘cause’ and what are the ‘effect’. For example, we often see upregulation of IL-6 in POI cases; however, it remains unclear if this is a primary event leading to follicular cell death or more of a secondary change? These mechanistic questions can be resolved with access to patient tissues and models, but using histological techniques (single-cell RNA sequencing, proteomics, metabolomics, etc.) will allow us to distinguish between primary and secondary changes.

Then, there is a local and systemic relationship that must be highlighted. In these patients, we often see changes in systemic inflammatory markers such as NLR ratio, C-reactive protein, etc., and the inflammatory milieu systemically can be playing a role by endocrine or immune pathways on the ovary; conversely, the hypoestrogenic state induced by ovarian failure can feed back to worsen systemic inflammation. Noting this local vs. systemic causality is critical to developing a holistic treatment strategy.

On the clinical translation front, well-designed clinical trials are needed to evaluate the efficacy and safety of various anti-inflammatory therapies in patients with POI. There is currently a lack of evidence from large samples that a specific anti-inflammatory therapy significantly improves fertility outcomes and/or endocrine function in patients with POI. The use of certain pharmaceutical agents, such as tacrolimus and talizumabs, in POI is reported from case reports and needs to be validated.

Because of the promise of stem cell transplantation and related interventions, it is important that their long-term safety and ethical implications be carefully studied and discussed. Patients with POI also often have fertility expectations; thus, any treatment approach should be tested for its effects on oocyte quality, embryo division, and ability to open up the fertility window.

In summary, inflammation plays a complicated pathologic role in POI, and POI is an important entry point for the understanding of inflammation and finding potential modulation targets. The promotion of using anti-inflammatory concepts in clinical treatment of POI should be based on in-depth mechanism analysis. The objective of future study is to find precise therapies targeting different inflammatory mechanisms by integrating different disciplines (reproductive endocrinology, immunology, inflammation biology, etc.) and models (multicell and multi-model validation). For example, for autoimmune POI, the application of individualized immunotherapy combined with short-term hormonal therapy may be attempted. For post-chemotherapy POI, applying anti-inflammatory and renal-protective herbs or small molecule protectors before or combined with primary treatment may be a possible option. For patients with idiopathic POI, the choice of anti-inflammatory therapy or other therapeutic methods may be based on the inflammatory hallmarks presented by the patients. The main goal is to restore ovarian function, delay or preserve fertility, and improve quality of life as much as possible while not compromising the patient’s interests. The validation and optimization of this approach is the best achieved by executing high-quality clinical studies. As the systematic understanding of inflammation’s role in POI develops, this goal will be realized.
